# Levels of selected analytes in the emissions of “heat not burn” tobacco products that are relevant to assess human health risks

**DOI:** 10.1007/s00204-018-2215-y

**Published:** 2018-05-05

**Authors:** Nadja Mallock, Lisa Böss, Robert Burk, Martin Danziger, Tanja Welsch, Harald Hahn, Hai-Linh Trieu, Jürgen Hahn, Elke Pieper, Frank Henkler-Stephani, Christoph Hutzler, Andreas Luch

**Affiliations:** 1 German Federal Institute for Risk Assessment (BfR), Department of Chemical and Product Safety, Berlin, Germany; 2 Official Chemical and Veterinary Surveillance Institute Sigmaringen, Sigmaringen, Germany

**Keywords:** Heat not burn, Smoke chemistry, Nicotine, Non-cigarette tobacco products, Carcinogens

## Abstract

Consumers of combustible cigarettes are exposed to many different toxicologically relevant substances associated with negative health effects. Newly developed “heat not burn” (HNB) devices are able to contain lower levels of Harmful and Potentially Harmful Constituents (HPHCs) in their emissions compared to tobacco cigarettes. However, to develop toxicological risk assessment strategies, further independent and standardized investigations addressing HPHC reduction need to be done. Therefore, we generated emissions of a commercially available HNB product following the Health Canada Intense smoking regimen and analyzed total particulate matter (TPM), nicotine, water, aldehydes, and other volatile organic compounds (VOCs) that are major contributors to health risk. We show that nicotine yield is comparable to typical combustible cigarettes, and observe substantially reduced levels of aldehydes (approximately 80–95%) and VOCs (approximately 97–99%). Emissions of TPM and nicotine were found to be inconsistent during the smoking procedure. Our study confirms that levels of major carcinogens are markedly reduced in the emissions of the analyzed HNB product in relation to the conventional tobacco cigarettes and that monitoring these emissions using standardized machine smoking procedures generates reliable and reproducible data which provide a useful basis to assess exposure and human health risks.

**Electronic supplementary material:**

The online version of this article (10.1007/s00204-018-2215-y) contains supplementary material, which is available to authorized users.

Tobacco consumption remains one of today’s major health hazards and was responsible for more than one in ten deaths in the year 2015 (GBD 2015 Tobacco Collaborators 2017). Consequently, tobacco control was strengthened by multiple measures in recent years, partly driven by implementation of the WHO Framework Convention on Tobacco Control (FCTC) (World Health Organization 2018). One strategy of tobacco companies to adapt to growing public and political pressure for further restrictions is the development of modified risk products or alternate tobacco products that are implied to be less hazardous. These claims are often based on reduced toxicant levels in the emissions, although these data cannot be directly translated into a health risk reduction. Notably, toxicant reduction strategies had also been proposed by WHO (World Health Organization 2014), opening discussions about feasibility of benefits for both smoking populations and individual smokers.

In principle, the conventional cigarettes are highly engineered products. A burning cigarette can be regarded as a connection of endo- and exothermic combustion systems (Baker et al. 2004). Yet, it gains complexity, since multiple mechanisms affect the generation of smoke (Muramatsu 2005). Smoke constituents are generated according to a temperature gradient depending on exothermic combustion within the burning tip. During puffs, temperatures can reach up to 950 °C. The majority of compounds, however, are formed in endothermic reactions within the adjacent pyrolysis-distillation zone where temperatures decrease from approximately 600 to 200 °C (Baker et al. 2004). Cigarette smoke consists of approximately 4800 compounds (Rodgman and Green 2003). At least 69 carcinogens had been identified by the year 2000 (Hoffmann et al. 2001) with an update to 98 hazardous components in 2011 (Talhout et al. 2011). Fowles and Dybing proposed an approach for prioritization of tobacco smoke constituents by applying toxicological risk assessment methods. They identified 1,3-butadiene and other substances like acetaldehyde as major contributors to cancer risk and thus suggested that harm reduction efforts should set a special focus on volatile organic compounds (Fowles and Dybing 2003).

Attempts to reduce the toxicity of tobacco smoke can be traced back to the 1960s. The initial strategies aimed for the reduction of specific compounds with ambiguous effects on overall toxicant levels (Baker et al. 2004). Further strategies to reduce toxicant levels included filter tips, filter perforation, as well as technical features such as porosity of cigarette paper and tobacco processing (Hoffmann et al. 2001). Although nicotine and tar content have decreased by more than 60% since the 1950s, this trend could not be linked to a drop in mortality rates among smokers. Furthermore, proliferation of low-yield cigarettes became a highly controversial issue. Despite the lower tar and nicotine contents, toxicant exposure has even increased when smoking intensities and profiles of long-term smokers are considered (Hoffmann et al. 2001). Further means to reduce the toxicity of tobacco smoke are limited, because combustion and consequently pyrolysis and distillation cannot be avoided in the conventional cigarettes. Since most hazardous compounds in tobacco smoke are formed between 200 and 700 °C, lower temperatures would limit formation of noxious compounds. Although earlier “heat not burn” (HNB) devices failed to gain consumer acceptance (Caputi 2016), these systems provide some advantages in terms of toxicant reduction compared to the conventional cigarettes (Henkler and Luch 2015).

First, in contrast to low-yield cigarettes, reduction of tar and associated toxicants is not necessarily interlinked with lower nicotine levels. Therefore, an increased consumption aimed at compensating deficient nicotine delivery becomes unlikely. Second, the previous reports indicate that far lower levels of relevant carcinogens can be achieved in newly developed HNB devices. One novel product referred to as “Tobacco Heating System 2.2” (THS2.2) is currently marketed in more than twenty countries. The manufacturer has stated that the yield of harmful and potentially harmful constituents (HPHC) is reduced by about 90% compared to the 3R4F reference cigarette. Importantly, a reduction of more than 95% was reported for major carcinogens, including benzene and 1,3-butadiene, when emissions were generated using the Health Canada Intense smoking regimen (Schaller et al. 2016).

From the perspective of risk assessment, it is essential to verify levels of toxicants including nicotine that can be reliably achieved in novel or modified tobacco products. It needs to be clarified whether standardized machine smoking procedures and standardized analytical methods lead to reproducible data that can be used to compare devices and to define a standard to be met if reductions were recognized as relevant. This is also an important prerequisite to address the issue of putatively modified health risks or to provide a differentiated risk assessment according to product features and specifications. However, independent investigations are scarce and urgently required. We have, therefore, analyzed the mainstream smoke emitted by THS2.2 products using different variants of commercially available tobacco sticks. This study was focused on the group of carcinogenic volatile organic compounds and aldehydes in particular according to the prioritization framework proposed by Fowles and Dybing (2003). The acquired data provide an important basis to address health risks and potential benefits in terms of a potentially reduced exposure to toxicologically relevant constituents.

Four tobacco heating devices and two different tobacco stick variants were analyzed with an LM4E smoking machine (Borgwaldt, Hamburg, Germany) following the Health Canada Intense smoking regimen (Health Canada 2000). Detailed description of analytical procedure can be found in the Supplementary Material. An overview of the measured levels of analytes in the emissions of the two different tobacco stick variants is given in Table 1. The obtained values from all used devices were pooled. We compared our emission findings to levels in mainstream smoke of different combustible cigarettes, including low and high tar, slim, and reference cigarettes, that were published by Counts et al. (2005). We displayed the lowest and the highest yields per analyte that could stem from different brands and calculated the corresponding reductions of our findings as averages of both stick variants. The levels of nicotine in this study were lower compared to the data provided by the manufacturer (Schaller et al. 2016) and also lower but still in the same range compared to the conventional cigarettes (Counts et al. 2005). Total particulate matter (TPM) was comparable to the manufacturer’s findings and higher than TPM from some combustible cigarettes. The yields of the carbonyl compounds formaldehyde, acetaldehyde, acrolein, and crotonaldehyde were, with a reduction of 80–96%, considerably lower when compared to combustible cigarettes (Table 1) and comparable to the published emissions observed by the manufacturer (Schaller et al. 2016). Similar to the carbonyl compounds, the emissions of the volatile and semi-volatile compounds benzene, 1,3-butadiene, isoprene, styrene, and toluene were with a reduction of 97 to over 99% markedly lower when compared to combustible cigarettes (Table 1). The range of values found is again similar to the manufacturer’s data (Schaller et al. 2016). To address consistency of nicotine and TPM release during the smoking procedure, the 12 puffs of the smoking protocol were divided into four intervals of three puffs each and analyzed separately. The nicotine and TPM release was shown to be inconsistent with lower yields in the beginning. More detailed information can be found in the Supplementary Material.

**Table 1:** Levels of analytes in the mainstream smoke of two different tobacco heating stick variants with “*n*” representing the number of replicates

Parameter	Unit	Stick variant 1		Stick variant 2		Combustible cigarettes (Counts et al. 2005)	Reduction
		Mean ± SD	*n*	Mean ± SD	*n*	Min–max (mean ± SD)	%
Puff count	Puff/stick	12 ± 0		12 ± 0		5.5 ± 0.3–13.6 ± 0.5	
TPM	mg/stick	52.6 ± 3.2	24	51.2 ± 3.2	24	27.5 ± 2.4–60.9 ± 3.3	
Nicotine	mg/stick	1.1 ± 0.1	24	1.1 ± 0.1	24	1.07 ± 0.06–2.70 ± 0.14	
Water	mg/stick	31.7 ± 5.5	24	28.5 ± 4.6	24	9.82 ± 1.42–21.35 ± 2.23	
NFDPM	mg/stick	19.8 ± 6.5	24	21.6 ± 5.9	24	16.3 ± 1.3–37.6 ± 2.1	
Acetaldehyde	µg/stick	179.4 ± 10.5	18	183.5 ± 10.1	14	930 ± 85–1540 ± 153	80.5–88.2
Acrolein	µg/stick	9.9 ± 1.2	18	8.9 ± 1.0	14	89.2 ± 7.3–154.1 ± 13.6	89.5–93.9
Formaldehyde	µg/stick	5.3 ± 0.4	18	4.7 ± 0.3	14	29.3 ± 3.8–130.3 ± 10.8	82.9–96.2
Crotonaldehyde	µg/stick	< 3.0	18	< 3.0	14	32.7 ± 1.5–70.8 ± 9.0	
1,3-Butadiene	µg/stick	0.22 ± 0.02	6	0.20 ± 0.02	6	77.0 ± 4.8–116.7 ± 14.3	99.7–99.8
Benzene	µg/stick	0.63 ± 0.07	6	0.54 ± 0.05	6	49.7 ± 7.7–98.3 ± 4.3	98.8–99.4
Isoprene	µg/stick	2.10 ± 0.35	6	1.82 ± 0.24	6	509 ± 41–1160 ± 65	99.6–99.8
Styrene	µg/stick	0.47 ± 0.06	6	0.49 ± 0.09	6	15.4 ± 0.8–33.3 ± 2.8	96.9–98.6
Toluene	µg/stick	2.15 ± 0.37	6	1.96 ± 0.23	6	86.2 ± 11.0–176.2 ± 15.7	97.6–98.8

Yields are compared to lowest and highest levels found by Counts et al. in combustible cigarettes

All levels were generated using HCI smoking regime

*TPM* total particulate matter, *NFDPM* nicotine-free-dried particulate matter

For a profound assessment of health risks and putative benefits, independent studies by different laboratories are needed. Furthermore, our intention was not only to reassess emissions of HPHC and compare to other studies, but also to use standardized methods as used by surveillance authorities and establish them for this particular application. More HNB products from different manufacturers are expected to appear on a wider market in the future with claims of reduced toxicant levels. Therefore, surveillance authorities will require standardized methods for routine analysis of HNB products to verify claims and to protect consumers from being misled.

In this study, we applied methods that are based on international standards to investigate emissions of a novel HNB product. We have used a commercially available linear smoking machine that was initially developed for electronic cigarettes. Thus, the procedure can be easily transferred. Our data are in good agreement with some recent investigations. In their recent study, Li et al. analyzed a set of HPHCs, including aldehydes and VOCs, in the emissions of the same HNB product using ISO and HCI smoking regimen (Li et al. 2018). The data presented in our study support their findings and conclusions. Farsalinos et al. analyzed the nicotine delivery in the preceding HNB model of the same manufacturer (Farsalinos et al. 2017). They found a higher nicotine yield as compared with the currently marketed THS2.2 that was analyzed here. Another study that used a custom instrument and custom smoking regimen reported similar findings for aldehydes but not for nicotine (Auer et al. 2017). A recent study by Bekki et al., that used the preceding HNB model as well, focused on tobacco-specific nitrosamines (Bekki et al. 2017). Their determined levels for nicotine, TPM, and water are comparable to ours. Another group developed a headspace solid-phase microextraction-based method for semi-quantitative assessment of VOCs emitted by HNB products (Savareear et al. 2017). The issue of toxicant reduction is complex, since these calculations depend on the reference product. Importantly, our data confirm absolute values for selected toxicants in the emissions of the analyzed HNB that are in agreement with data published by the manufacturer (Schaller et al. 2016). Furthermore, our study is in agreement with the currently published FDA Tobacco Products Scientific Advisory Committee (TPSAC) briefing document (Food and Drug Administration 2018).

Another interesting point to show was that emissions of particulate matter and nicotine were not consistent during the smoking procedure. Unlike electronic cigarettes, in the European Union conventional cigarettes are not regulated to provide consistent nicotine delivery. Although HNB products are likewise not regulated in terms of consistency of nicotine delivery, the observed inconsistent delivery may influence consumer satisfaction, nicotine blood levels, and adaptations of smoking behavior, and needs to be investigated further.

In our study, we found comparatively high levels of tar. For the conventional cigarettes, “tar” is defined as particulate matter subtracted by nicotine and water (ISO 4387:^2000^), and is limited to 10 mg tar per cigarette as determined with the ISO smoking regimen (ISO 3308:^2012^) according to European regulations (EU 2014). Importantly, the water content in the smoke of the HNB product is high compared to the conventional cigarettes, thus affecting the NFDPM calculation more than in the conventional cigarettes. The manufacturer applied a special instrumental set-up to avoid the loss of water (Ghosh and Jeannet 2014). This special equipment is neither standardized nor applicable for surveillance authorities. Therefore, we decided to use the extraction and titration method which is already applied in routine analysis.

Although the NFDPM value for HNB products can be formally calculated as for the conventional cigarettes, direct comparisons would be misleading. TPM of the conventional cigarettes, which is defined as the portion that is trapped on the filter (ISO 4387:^2000^), contains typical toxicants that were confirmed to be strongly reduced in the analyzed HNB product. In contrast, the proportion of humectants in NFDPM of HNB products is markedly higher compared to the conventional tobacco cigarettes.

The strongly reduced HPHC levels in the emissions of the analyzed HNB device are likely to reduce toxicant exposure. Nevertheless, it should be noted that machine smoking protocols are standardized methods aimed to monitor reliable emissions, but not accurate models for human exposure or smoking behavior. Further studies are required to address the magnitude of exposure reduction. However, the herein confirmed reductions of relevant toxicants by about 80–99% are substantial, leading to the relevant question of putatively reduced health risks. Risk assessment models need to be established that could take advantage of the framework for prioritization of carcinogens in cigarette smoke as proposed by Fowles and Dybing (2003). Mainstream smoke constituents were prioritized according to their concentrations and their cancer potency factors. A recent study performed calculations with one data set of THS2.2 and provisionally concluded cancer potencies of HNB products to be more than 10% lower than the conventional cigarettes (Stephens 2018). We could confirm a highly substantial reduction of prioritized major carcinogens, such as 1,3-butadiene, acetaldehyde, and benzene. Several studies addressed lowered health risks due to reduced smoking of tobacco cigarettes and substantial data are available (Inoue-Choi et al. 2018; Law et al. 1997; Pesch et al. 2012). It is still uncertain whether these data are applicable to model reduced exposure in relation to HNB products. Although modified health risks are expected, it is difficult to provide an estimate for both populations and individual smokers.

HNB products are a novelty to the market and more manufacturers are expected to launch new versions in this product category. Therefore, it is essential to define criteria that should be met by new products. Analytical assessment of HPHC contents in mainstream smoke can help to define these standards. Regarding a risk–benefit analysis that is required for novel tobacco products in Europe (2014/40/EU) (EU 2014), substantial reductions of toxicant levels might be regarded as a discrete benefit compared to combustible cigarette consumption, even if potential consequences for human health still need to be explored. This is consistent with the previous approaches proposed for the conventional cigarettes by WHO (World Health Organization 2014).

We propose that new HNB products need to show comparable or lower HPHC levels in the emissions as the analyzed device to confirm a benefit in the context of an overall risk assessment. The applicable values for toxicant levels should be continuously minimized and reassessed when refined products and technologies become available. By contrast, it should be considered insufficient to show only a minor decrease of HPHC levels in comparison to the conventional cigarettes. Furthermore, it should be assessed whether other levels of toxicologically relevant substances are elevated in return as already discussed for propylene glycol, glycerol, glycidol, and acetol (Food and Drug Administration 2018). Therefore, further studies need to be conducted: first, more independent assessments of toxicant yields need to be published by using standardized methods for the above discussed reasons. Second, it should be examined whether HNB products lead to other toxicants and health hazards that have been neglected so far. Finally, the long-term impact on public health needs to be assessed in the future.

## Electronic supplementary material

Below is the link to the electronic supplementary material.

Supplementary material 1 (DOCX 520 KB)

## Abbreviations

FCTC: Framework convention on tobacco control
FDA: Food and Drug Administration
HNB: Heat not burn
HPHC: Harmful and Potentially Harmful Constituents
ISO: International Organization for Standardization
NFDPM: Nicotine-free-dried particulate matter
THS2.2: Tobacco Heating System 2.2
TPM: Total particulate matter
VOCs: Volatile organic compounds
WHO: World Health Organization
