# Supplementary material for: Levels of selected analytes in the emissions of “heat not burn” tobacco products that are relevant to assess human health risks
Source: Arch Toxicol. 2018 May 5;92(6):2145–9. doi: 10.1007/s00204-018-2215-y (PMC6002459; doi:10.1007/s00204-018-2215-y)
Supplement: Supplementary file 1 — Supplementary material 1 (DOCX 520 KB) [file 204_2018_2215_MOESM1_ESM.docx]

**Supplementary Material**

1. Materials and Methods

**1.1. Tobacco heating devices and tobacco sticks**

Tobacco heating devices (Smith et al., 2016) and two variants of the corresponding tobacco sticks containing different tobacco blends were purchased at local stores in Berlin, Germany. The heating devices were cleaned according to the recommendations of the manufacturer after every 18 runs using the provided cleaning sticks. The tobacco sticks were conditioned at 22 ± 1°C, 60 ± 2% rH for at least 48 hours (ISO 3402, 1999). Four heating devices with different usage histories were used as displayed in Supplementary Table 1.

**Supplementary Table 1.** Overview on the different tobacco heating devices and their history of usage prior to this study.

| Device | History of usage |
| --- | --- |
| Device I | Unused |
| Device II | Used by a consumer for approximately one month and tested in a smoking machine with about 80 tobacco sticks |
| Device III | Applied in a smoking machine (about 60 tobacco sticks) |
| Device IV | Applied in a smoking machine (about 60 tobacco sticks) |

**1.2. Chemicals and standard substances**

All chemicals and solvents were of analytical grade or higher. Acetonitrile and orthophosphoric acid (85%) were purchased from Merck KGaA (Darmstadt, Germany) and methanol from Merck Millipore (Billerica, MA, USA). 2,4-Dinitrophenylhydrazine (moistened with 33% water) was obtained from PanReac AppliChem (Darmstadt, Germany), hydranal solution from Honeywell Fluka (Hydranal-Composite 5, Morris Plains, NJ, USA). 2‑Propanol containing the internal standards ethanol (2 g/L) and n-heptadecane (0.3 g/L) was bought from LGC Standards (Teddington, UK), and tris(hydroxymethyl)aminomethane from Sigma-Aldrich (St. Louis, MO, USA). Carbon dioxide for the generation of dry ice was obtained from Air Liquide (Paris, France). Analytical standards for benzene (99.96%), benzene-d_6_ (99.96%), 1,3-butadiene (99.5%), isoprene (>99.5%), styrene (99.9%), toluene (99.9%) and the 2,4-dinitrophenylhydrazone (DNPH) derivatives of the carbonyl compounds acetaldehyde (99.9%), acrolein (99.5%), crotonaldehyde (99.9%) and formaldehyde (99.9%) were purchased from Sigma-Aldrich (St. Louis, MO, USA). (*S*)-nicotine salicylate (99.8%) was purchased from LGC Standards (Teddington, UK).

**1.3. Generation of mainstream smoke**

The mainstream smoke of tobacco sticks was generated using an LM4E smoking machine (Borgwaldt, Hamburg, Germany) with a PM1 piston pump unit (Borgwaldt, Hamburg, Germany) applying the Health Canada Intense smoking regimen (Health Canada, 2000) in order to maximize the number of drawn puffs. Tobacco heating devices were activated by button pushing for 3 s followed by a 20 s heating interval before the first puff was taken. Puff volumes of 55 mL were drawn within 2 s (puff duration) at a frequency of 30 s. Due to the device heating time of 6 min, a maximum of 12 puffs were taken. After the heating was switched off automatically, an additional clearing puff was performed. Differing from the HCI protocol, filter tips of the tobacco sticks were not covered with tape.

**1.4. Determination of total particulate matter (TPM), nicotine, water and nicotine-free dry particulate matter (NFDPM) per tobacco stick and TPM and nicotine per three puffs**

Mainstream smoke of three tobacco sticks was collected on a Cambridge glass-fiber filter pad (Ø 44  mm, Borgwaldt Köber Solutions, Hamburg, Germany). After gravimetric determination of TPM (CP 225D-0CE, Sartorius, Göttingen, Germany) the glass-fiber filter pad was extracted with 50 mL of isopropanol containing the internal standard n-heptadecane (0.3 g/L) on a shaker (SM-30 Control, Edmund Bühler , Hechingen, Germany) for at least 30 min at 60 rpm. The water content was analyzed with Karl-Fischer titration (841 Titrando, 803 TiStand, Metrohm, Filderstadt, Germany) using 5 mL of the extract and two to three titrations per sample. Nicotine was quantified by gas chromatography applying flame ionization detection at 300.0°C (7890A, Agilent Technologies, Santa Clara, CA, USA; 30 mL/min H_2_ flow, 99.999%; 400 mL/min air flow; 15 mL/min make up flow, N_2,_ 99.999%; Air Liquide, Paris, France) on an HP-5 column (30 m x 0.530 mm, 2.65 µm film, Agilent Technologies, Santa Clara, CA, USA). 1.0 µL of extract was injected in splitless mode at the injection temperature of 250.0°C. The flow rate of the carrier gas helium (99.999%, Air Liquide, Paris, France) was 5.50 mL/min. The oven was programmed with the following temperatures: hold at 120.0°C for 5 min; a linear increase to 230.0°C for 6 min; hold at 230.0°C for 5.5 min. Nicotine and water contents were subtracted from the TPM to calculate nicotine-free dry particulate matter (NFDPM). For each combination of the four devices and two tobacco stick variants, six replicates were analyzed.

To examine the continuity of nicotine release into the mainstream smoke four intervals comprising three individual puffs (12 puffs per stick in total) were analyzed. The mainstream smoke for each interval was collected on separate glass-fiber filter pads per interval. TPM was determined gravimetrically. The mainstream smoke of the respective interval of three tobacco sticks was combined on the same filter for subsequent nicotine analysis: The glass-fiber filter pads were extracted with 20 mL of isopropanol containing 0.3 g/L n-heptadecane. Nicotine was quantified as mentioned above.

**1.5. Determination of carbonyl compounds**

The mainstream smoke of three tobacco sticks was not filtered by a Cambridge glass-fiber filter pad, but directly carried through two impingers containing 35 mL 2,4-dinitrophenylhydrazine solution in a row. After 30 min derivatization time, 8 mL of the sample solution was stabilized with 2 mL tris(hydroxymethyl)aminomethane solution (16 mg/mL). For devices I and II, five replicates were generated for each tobacco stick variant while four replicates were generated for devices III and IV.

DNPH derivatives of formaldehyde, acetaldehyde, crotonaldehyde and acrolein were quantified by liquid chromatography (1100 series: binary pump G1312 A, degasser G1312 A, column oven G1312 A, autosampler G1312 A, Agilent Technologies, Santa Clara, CA, USA) coupled to diode array detection at 360 nm (DAD G1312 A, Agilent Technologies, Santa Clara, CA, USA) and equipped with an RP-Amid column (Ascentis, 150 x 2 mm, 3 µm, Supelco, Bellefonte, PA, USA). The separation was carried out at 20°C and a gradient elution with water (eluent A) and acetonitrile (eluent B) using the following gradient: 0 - 10 min, a linear gradient from 40% to 50% B; hold for 4 min; 14 - 26 min, a linear gradient to 80% B; hold for 2 min. The flow rate was 300 µL/min. Injection volume was 20 µL. All analytes were qualified by comparing the retention time to standards. Acetaldehyde-DNPH, crotonaldehyde-DNPH and acrolein-DNPH were also qualified by their UV spectra. The presence of all analytes in the sample was confirmed by LC-MS/MS (binary pump G1312 A, degasser G1379 B, column oven G1316 B, Agilent Technologies, Santa Clara, CA, USA; autosampler PAL HTS, PAL Systems, CTC Analytics AG, Zwingen, Switzerland; mass spectrometer API 4000, Sciex, Framingham, MA, United States) in negative mode with two transitions per analyte: m/z = 209 to 151 and 120 for formaldehyde, m/z = 223 to 151 and 76 for acetaldehyde, m/z = 235 to 181 and 158 for acrolein and m/z = 249 to 181 and 163 for crotonaldehyde.

**1.6. Determination of volatiles and semi-volatiles**

Mainstream smoke of nine tobacco sticks filtered by a Cambridge glass-fiber filter pad was carried through two impingers containing 10 mL of methanol in a cold trap. The temperature of the cold trap was held below –70°C with dry ice and isopropanol. Afterwards both sample solutions were spiked with 400 µg benzene-d_6_ each and combined. Samples were stored in closed microvials at –20°C. For devices I and II three replicates were generated per tobacco stick variant. All samples were injected and analyzed in duplicate.

Quantification of 1,3-butadiene, benzene, isoprene, styrene and toluene was performed using an Agilent HP 6890 gas chromatograph (Agilent Technologies, Santa Clara, CA, USA), equipped with an injector 7683 (split/splitless, Agilent Technologies, Santa Clara, CA, USA) and an HP-Plot Q column (30 m x 0.32 mm, 20 µm film, Agilent Technologies, Santa Clara, CA, USA) and a mass spectrometer Agilent MSD 5973. A helium gas flow of 7.0 mL/min was used with the following temperature program: hold 150°C for 5 min; linear increase to 170°C at 5°C/min; linear increase to 220°C at 20°C/min and hold for 1.5 min; linear increase with 5°C/min up to 260°C and final hold for 4 min. 1 µL of each sample was automatically injected in split mode with a split ratio of 5:1 and an inlet temperature of 250°C. The temperatures of the electron ionization ion source and the quadrupole were 230 and 180°C, respectively. Acquisition was performed in selective ion monitoring (SIM) mode, with the following five groups: start at 3.0 min the m/z ratios 27, 39, 50, 51, 53, 54 with dwell times of 40 ms; after 6.0 min the m/z ratios 26, 39, 51, 52, 53, 67, 68 with dwell times of 32 ms; after 10.0 min with the m/z ratios 50, 56, 77, 78, 82, 84 with dwell times of 40 ms; after 14.0 min the m/z ratios 39, 65, 91, 92 with 67 ms dwell time; after 18.0 min the m/z ratios 77, 78, 103, 104 and 67 ms dwell time.

2. Results in detail

**2.1. Carbonyl compounds**

The yields of the carbonyl compounds formaldehyde, acetaldehyde, acrolein and crotonaldehyde were determined (Supplementary Figure 1). Mainstream smoke was generated with four different heating devices and two tobacco stick variants. During our experimental procedure, device IV stopped working without noticeable reason. Thus the data set with this device could not be completed. Due to insufficient baseline separation, levels of crotonaldehyde were only assessed semi-quantitatively and are not presented in Figure 1. The threshold was set at 3.0 µg/stick and all samples resulted in concentrations below the threshold.

**2.2. Volatile and semi-volatile compounds**

The yields of the volatile and semi-volatile compounds benzene, 1,3-butadiene, isoprene, styrene and toluene are illustrated in Supplementary Figure 2. Each bar represents one device and one tobacco stick variant. Only the newest (I) and the oldest (II) device have been used to address this issue.

**2.3. Inconsistent release of nicotine in the initial puffs**

Each tobacco stick was smoked with 12 puffs. These 12 puffs were divided into four intervals of three puffs each: puffs 1 to 3 (interval 1, I1); puffs 4 to 6 (interval 2, I2); puffs 7 to 9 (interval 3, I3) and puffs 10 to 12 (interval 4, I4). Therefore, I1 represented the beginning of the smoking procedure whereas I4 resembled the end. As shown in Supplementary Figure 3, the TPM yields for both variants and for all four devices are the highest in the beginning of the smoking procedure (I1 with 14 mg and I2 with 14.8 mg as the mean of both variants) and then decrease during the second half of the smoking process with a minimum yield at the end (I4 with 8.8 mg for both variants). The dispersion (n=4) is also higher for I1 and I2 than for I3 and I4, indicating that the variability of the TPM yield is lower in the second half of the smoking procedure. However, the nicotine yield shows a minimum in the beginning (I1) and a maximum in the middle of the smoking process (I2 and I3). At the end it decreases slightly (I4). Nicotine levels were initially lower than 50% of the levels found in the middle of the smoking procedure and therefore represent only 10-12% of the total nicotine yield.

3. References

Health Canada (2000). "Tobacco Reporting Regulations, SOR/2000-273".).

ISO 3402 (1999). "Tobacco and tobacco products - atmosphere for conditioning and testing". International Organisation for Standardisation, Geneva).

Smith, M.R., Clark, B., Ludicke, F., Schaller, J.P., Vanscheeuwijck, P., Hoeng, J., et al. (2016). Evaluation of the Tobacco Heating System 2.2. Part 1: Description of the system and the scientific assessment program. Regul Toxicol Pharmacol 81 Suppl 2, S17-S26. doi: 10.1016/j.yrtph.2016.07.006.





**Supplementary Figure 1.** Yields of the carbonyl compounds acetaldehyde (A), acrolein (B) and formaldehyde (C) generated with two different stick variants and the devices I, II, III and IV. Determination of crotonaldehyde was semi-quantitative and is therefore not displayed. There were 5 measurements (repeats) for devices I and II, and 4 for devices III and IV. Device IV stopped working before stick variant 2 could be assessed.





**Supplementary Figure 2.** Levels of the volatile compounds 1,3-butadiene (A), benzene (B), isoprene (C), styrene (D), and toluene (E) generated with two different stick variants. A new device (I) and the device with the longest usage history in this study (II) were used. There were 3 measurements (repeats) with double determination each.





**Supplementary Figure 3.** For machine smoking of one tobacco stick 12 puffs were conducted. These 12 puffs were divided into four intervals of three puffs each: interval 1 with puffs 1 – 3, interval 2 with puffs 4 – 6, interval 3 with puffs 7 – 9, and interval 4 with puffs 10 – 12. Stick variant 1 (A, C) and variant 2 (B, D) were smoked with the devices I, II, III, IV. For TPM (A, B) determination the number of repeats was 12, for nicotine (C, D) 4 with the exception of device III with variant 1 (C). Here only 3 repeats were performed.
